# Trend of Serum CEA in Recurrent Signet Cell Colorectal Adenocarcinomas

**DOI:** 10.1007/s13193-025-02340-6

**Published:** 2025-05-23

**Authors:** Preeti Vijayakumaran, Mufaddal Kazi, Ashwin Desouza, Avanish Saklani

**Affiliations:** 1 Department of Surgical Oncology, Tata Memorial Hospital, Mumbai, India; 2 Department of Gastrointestinal and HPB Oncology, Tata Memorial Hospital, Mumbai, Maharashtra India

**Keywords:** CEA, Colorectal cancer, Signet cell

## Abstract

Colorectal cancer (CRC) represents a major global health burden, with signet cell adenocarcinoma constituting a rare but aggressive subtype. Despite multimodality treatment with curative intent, recurrence rates remain significant, and outcomes are poor. Carcinoembryonic antigen (CEA) is a widely used biomarker for CRC follow-up; however, its role in signet cell CRC remains inadequately defined. This study aimed to evaluate the trends in serum CEA among baseline CEA secretors and non-secretors presenting with recurrence after curative treatment and assess its value in postoperative surveillance. This retrospective study analyzed data from a prospectively maintained database at a tertiary cancer center between June 2011 and October 2021. Inclusion criteria were patients with recurrent signet cell colorectal adenocarcinoma, treated with curative intent, and with available CEA values at baseline, treatment completion, and recurrence. Variables included demographic data, baseline CEA levels, recurrence patterns, and CEA status at recurrence. Baseline CEA secretors were defined as those with preoperative CEA > 5 ng/ml. Statistical analysis employed chi-square and Fisher’s exact tests for categorical data, with significance set at *p* < 0.05. Out of 263 signet cell colorectal adenocarcinoma patients, 100 recurrent cases were analyzed. Baseline CEA secretors accounted for 35%, while 65% were non-secretors. Elevated CEA levels at recurrence were observed in 94.3% of baseline secretors and 67.7% of non-secretors. Among secretors, only 5.7% showed normal CEA at recurrence. Recurrence patterns revealed no significant correlation with baseline secretor status, though peritoneal recurrences were more frequent among secretors. Most recurrence cases, irrespective of baseline CEA levels, exhibited elevated CEA levels, emphasizing its relevance in surveillance. This study highlights the importance of CEA monitoring in the follow-up of recurrent signet cell colorectal adenocarcinoma. Elevated CEA levels are a reliable marker for recurrence, even in baseline non-secretors. Conversely, normal CEA in secretory patients offers a reassuring prognostic indicator. The study highlights the non-site-specific nature of CEA elevation at recurrence. The study’s findings support the continued use of serial CEA measurements in the postoperative surveillance of signet cell CRC.

## Introduction

Colorectal cancer (CRC) stands as a formidable global health challenge, ranking third in the incidence of cancer and second in cancer-associated mortality worldwide. While conventional adenocarcinomas comprise the majority of CRC cases, primary colorectal signet cell carcinoma emerges as a distinctive and aggressive variant, impacting predominantly younger individuals and constituting approximately 1–2% of all colorectal malignancies [1, 2].

A recent audit from a tertiary care center in India has highlighted a notable prevalence of signet cell-type CRC, accounting for up to 13% of cases [3]. Alarming recurrence rates, ranging between 35 and 40% following curative resection of locally advanced colorectal cancer, underscore the urgent need for improved surveillance strategies in this population, with a median recurrence-free survival of 15 months [4].

Postoperative follow-up plays a crucial role in managing CRC, aiming to detect tumor recurrence promptly and facilitate timely intervention, thereby potentially enhancing patient survival outcomes.

Carcinoembryonic antigen (CEA) is an important prognostic biomarker used in monitoring treatment and predicting the recurrence of colorectal cancer [5, 6]. Baseline CEA secretors (CEA level above 5 ng/ml) are in 20–30% of colorectal cancers [7]. The sensitivity and specificity of CEA in predicting postoperative recurrence range between 60–80% and 66–98%, respectively [8, 9]. Preoperative CEA levels influence post-curative resection CEA dynamics [10]. CEA elevation in recurrence may also be seen in patients with normal preoperative CEA. Hence, serial postoperative CEA testing cannot be discarded based on a normal preoperative serum CEA [11].

Despite its utility, controversies persist regarding the comprehensive role of CEA in colorectal cancer management. While certain studies suggest its predictive value in assessing resectability and survival, others question its efficacy in detecting early recurrence [12, 13]. The precise impact of CEA on mortality outcomes remains to be proven [14]. Nonetheless, even in non-secretors, CEA surveillance is routinely recommended and practiced, albeit its value remains incompletely understood.

CEA as a surveillance marker in signet cell CRCs has not been well studied. Signet ring cancers have a higher risk of recurrence. The time from recurrence to death is short. This leads to anxiety in both patients and surgeons. These tumors are not PET avid and are assumed to be non-secretors.

We aim to understand the role of CEA surveillance in signet cell colorectal cancers and to understand the negative predictive value of a normal CEA value during the follow-up of these patients.

## Methods

The study was conducted in the colorectal oncology unit of our tertiary referral cancer center. It was designed as a retrospective, observational study from a prospectively maintained database, covering the period from June 2011 to October 2021. The inclusion criteria comprised patients with recurrent signet ring cell colorectal cancers, treated with curative intent, with CEA values available at presentation, treatment completion, and recurrence, and with known sites of relapse. Patients with metastatic disease at presentation, those who defaulted or progressed during treatment, or those without available CEA status at baseline, treatment completion, or follow-up were excluded.

The variables recorded included basic demographic data, baseline CEA levels, recurrence, the site of recurrence, and CEA levels at recurrence. Elevated CEA was defined as a value above 5 ng/ml on a single occasion. Right-sided colon cancer was categorized as cancer of the cecum, ascending colon, hepatic flexure, and transverse colon. In contrast, cancer of the splenic flexure, descending colon, and rectosigmoid were considered left-sided colon cancer. Rectal cancer was defined as cancer up to 15 cm from the anal verge. The presence of any signet cells in pathology specimens was classified as a signet cell variant according to institutional practice.

The study’s primary objective was to examine the change in CEA secretory status among baseline secretors and non-secretors of recurrent signet cell colorectal cancer patients. The secondary objectives were to study the site of recurrence and the correlation between the site of recurrence and CEA status.

Data were analyzed using SPSS version 22.0. Continuous variables were summarized as median, range, and 95% confidence intervals (CIs). Categorical variables were compared using the chi-square test or Fisher’s exact test, while multivariate analysis was conducted using ANOVA. A *p*-value of < 0.05 was considered statistically significant.

Colon cancer was operated on with or without peri-operative therapy according to the stage and standard guidelines. Most patients of rectal cancer were offered neoadjuvant chemoradiation (long course/short course), followed by surgery, followed by adjuvant therapy. All the patients were treated as per the standard of care after the institute’s multidisciplinary team evaluation.

Postoperative surveillance was conducted in accordance with institutional protocols. Serum CEA levels were assessed every 3 months during the first 2 years, followed by 6-month evaluations until 5 years. Imaging with contrast-enhanced CT (CECT) of the thorax, abdomen, and pelvis was performed annually in the first 3 years and then at the completion of 5 years Colonoscopy was done at baseline/treatment completion, followed by once in 5 years for normal-risk individuals. A PET scan was recommended for patients seen on follow-up with two elevated CEA levels at 6-week intervals with no evidence of structural disease on the CT scan.

## Results

**Fig. 1:**
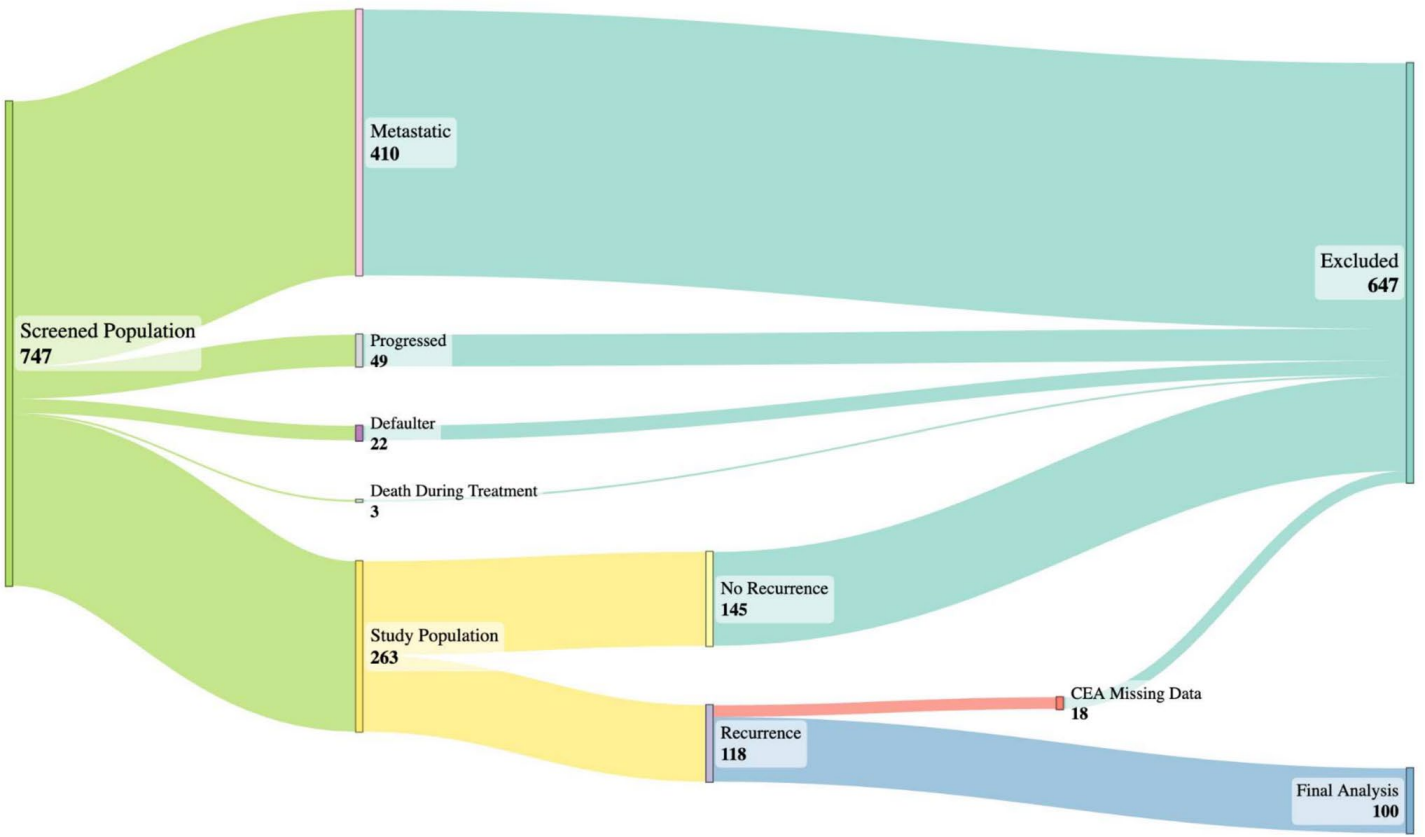
Sankey diagram of study population

In this study, 747 patients with signet ring cell colorectal cancer presenting to our tertiary care center were screened, of whom 263 were included in the analysis, as outlined in Fig. 1. Disease recurrence was observed in 118 of these 263 patients (44.86%). After excluding 18 patients with missing CEA values, 100 recurrent cases were included in the final analysis. Baseline characteristics of these patients are presented in Table 1. The mean baseline CEA level was 3.75 ng/mL, and the median follow-up duration was 14 months.

**Table 1:** Baseline characteristics of all 100 patients of recurrent signet cell colorectal adenocarcinoma

Characteristics		All patients (100)	Secretors (35)	Non-secretors (65)	*P* value
Age (years)	Median	36 (18–71)	33 (18–66)	37 (18–71)	0.656
Female	%	28 (28%)	9 (27.3%)	19 (29.2%)	0.451
Male	%	72 (72%)	26 (78.8%)	46 (70.7%)	
Mucinous histology	Yes	38 (38%)	14 (40%)	24 (37%)	0.762
	No	62 (62%)	21 (60%)	41 (63%)	
Stage	I	0	0	0	0.336
	II	8 (8%)	1 (2.9%)	7 (10.8%)	
	III	83 (83%)	30 (85.7%)	53 (81.5%)	
	IV	9 (9%)	4 (11.4%)	5 (7.7%)	
Locations	Right colon	10 (10%)	4 (11.4%)	6 (9.2%)	0.468
	Left colon	5 (5%)	2 (5.7%)	3 (4.6%)	
	Rectum	85 (85%)	29 (82.9%)	56 (86.2%)	
Preoperative therapy	Yes	84 (84%)	28 (80%)	56 (86.2%)	0.327
	No	16 (16%)	7 (20%)	9 (13.8%)	
Adjuvant chemotherapy	Yes	87 (87%)	33 (94.3%)	54 (83.1%)	0.128
	No	13 (13%)	2 (5.7%)	11 (16.9%)	
Follow-up (months)	Median	14	11	19	
Deaths	Yes	30	7 (20%)	23 (35.3%)	0.109
	No	70	28 (80%)	42 (64.6%)	

Among the final cohort of 100 patients, 35% were baseline secretors, while 65% were non-secretors. The recurrence rate in the 263 patients of the study population was 43.5% among secretors and 43.03% among nonsecretors(not statistically significant; *p* = 0.939). In total, 94.3% (33 out of 35) of baseline secretors and 67.67% (44 out of 65) of baseline non-secretors exhibited CEA elevation at recurrence; 5.7% (2 out of 35) of the baseline secretors did not manifest elevated CEA levels at recurrence, as depicted in Table 2.

**Table 2:** Baseline secretor status and CEA at recurrences

	Baseline secretor	Baseline non-secretor	Total
CEA > 5 at recurrence	33 (94.3%)	44 (67.7%)	77 (77%)
CEA </= 5 at recurrence	2 (5.7%)	21 (32.3%)	23 (23%)
Total	35	65	100

Among baseline secretors, 22.9% of patients experienced only local recurrence, 31.4% peritoneal recurrence, 31.4% systemic recurrence, and 14.2% peritoneal and other systemic recurrences. In comparison, among non-secretors, 18.5% encountered only local recurrence, 23.1% peritoneal recurrence, 43.1% systemic recurrence, and 15.3% peritoneal and other systemic disease (Table 3). Peritoneal recurrences were more frequently observed among baseline secretors. All peritoneal recurrences among secretors were accompanied by elevated CEA levels at recurrence. Regardless of baseline CEA status, most patients, comprising 76.7%, 78.1%, and 74.1% with local, peritoneal, and systemic recurrences, presented with elevated CEA levels at recurrence.

**Table 3:** Site-wise distribution of recurrences

Site of recurrence	Secretor (35)		Non-secretor (65)	
	CEA > 5 at recurrence (33)	CEA </= 5 at recurrence (2)	CEA > 5 at recurrence (44)	CEA </= 5 at recurrence (21)
Local/pelvic	8	0	7	5
Peritoneal	11	0	10	4
Systemic	7	1	16	6
Local + peritoneal	0	0	1	0
Peritoneal + systemic	5	0	5	5
Local + systemic	2	1	5	1

Table 3 describes the site-wise distribution of recurrences. The sites of recurrences are categorized as local, peritoneal, or systemic. Local recurrence in rectal cancer is confined to the pelvis (presacral, para-rectal, bladder trigone) and iliac nodes. Local recurrence in the colon is defined as operative bed recurrence.

## Discussion

Signet ring histology in the Indian subcontinent accounts for more than 10% of all colorectal cancer patients compared to less than 1% in the Western world [3, 15]. The outcome remains dismal, with high recurrence rates and early demise after the detection of recurrence [2]. The role of PET/CT scans and CEA surveillance in this subgroup is uncertain.

Our study shows that despite a low secretory state at baseline, CEA on follow-up of signet ring cancers remains essential, with 67% of non-secretors having rising CEA at recurrence. On the other hand, a negative CEA in secretory patients is reassuring to both patients and doctors, as less than 3% of patients with normal CEA recur.

In colorectal cancer in general, intensive surveillance protocols following curative resection have demonstrated associations with improved overall survival rates and increased resection rates for recurrent disease, particularly by enhancing the detection of asymptomatic recurrences amenable to surgical intervention [16].

Carcinoembryonic antigen (CEA), discovered by Gold et al. in 1965, is an important prognostic biomarker for monitoring treatment and predicting recurrence in colorectal cancer (CRC) [17]. Elevated preoperative CEA levels are associated with an increased relative risk of recurrence [18].

CEA is pivotal in detecting asymptomatic recurrence, accounting for approximately 63% of recurrent CRC cases. In patients with elevated CEA levels but no apparent structural disease on PET/CECT imaging, a CEA threshold of 10 ng/ml and a 1.36-fold increase in CEA value over 3 months significantly predict future recurrence, with respective sensitivities and specificities of 80% and 70% [19].

Preoperative CEA levels influence CEA levels post-curative resection and recurrence rates. Among individuals with baseline CEA levels exceeding 5 ng/ml (secretors), a return to baseline levels post-resection correlates with prolonged time to recurrence. In a retrospective analysis conducted by Holt et al., encompassing 186 patients diagnosed with colorectal cancers (CRCs), 21.5% of the cohort were initial secretors. Among baseline secretors, 66% exhibited elevated levels of CEA at recurrence, while 50% of non-secretors showed a similar elevation. This study revealed a significant association between CEA elevation and CRC recurrence, irrespective of the patient’s initial secretor status [20]. In another study by Chong et al., in 699 cases of colorectal cancer recurrence, baseline secretors constituted 49.5%, and 50.5% were non-secretors. During follow-up, all non-secretors and 58% of secretors achieved a CEA < 5 µg/L. A new rise in CEA to > 5 µg/L prior to relapse was observed in 51% of all patients. This new rise was more likely to be observed among patients whose initial primary was secretory (*p* < 0.01) [10]. Preoperative CEA levels prior to initial surgery influence CEA dynamics post-curative resection in patients with recurrent colorectal cancer.

Our study was derived from the observation that many signet cell colorectal cancers presented with an elevated CEA at recurrence, irrespective of pretreatment CEA values. With the present cohort, we aimed to understand the pattern of CEA and the sites of relapses in recurrent signet ring cell colorectal cancers.

The role of CEA secretor status and correlation with recurrence has not been studied in signet cell colorectal adenocarcinoma patients. With our investigation, we endeavor to explore the non-secretor to secretor transformation rate in recurrent signet cell colorectal cancers.

Within our study cohort, comprising non-metastatic signet cell colorectal cancer patients, we observed that 32% exhibited baseline carcinoembryonic antigen (CEA) secretory state, defined as CEA levels exceeding 5 ng/ml.

Our findings elucidate a prevalent occurrence of CEA elevations in most recurrent cases, regardless of the baseline secretor status. Specifically, 94.3% of baseline secretors and 67.7% of non-secretors manifested CEA elevation upon recurrence. A small subset of baseline secretors (5.7%) did not display elevated CEA levels at recurrence. Conversely, a substantial proportion of non-secretors presented with elevated CEA at recurrence (much more than the previously reported trials on all colorectal cancers), emphasizing its relevance in surveillance, irrespective of baseline CEA values, even in signet cell colorectal cancers.

Moreover, we investigated the association between CEA secretor status and the site of recurrence (local, peritoneal, systemic), revealing no significant correlation. This result suggested a non-site-specific nature of CEA elevation. This result correlated with a study by Shin et al., where no differences between the different CEA groups were noted regarding the site of recurrence in terms of locoregional and distant metastasis.

Although our study contributes valuable insights, certain limitations warrant acknowledgement. Specifically, our inclusion criteria encompassed all patients with histological evidence of signet cells, regardless of the proportion, deviating from the WHO definition requiring > 50% presentation of signet cells histologically. Additionally, while our sample size surpasses previous studies in recurrent signet cell CRCs, it remains modest. Furthermore, limited documentation of cases in the early years and unavailable CEA trends at recurrence for most cases constitute notable limitations.

In essence, our study underscores the diagnostic importance of CEA levels at recurrence in signet cell colorectal cancer, reinforcing the routine assessment of CEA in follow-up evaluations for this subgroup of patients, regardless of baseline secretor status.

## Conclusions

Our study was conducted to understand the role of surveillance of CEA in the signet cell variant of colorectal cancer.

In conclusion, our findings reveal that despite the low secretory state at baseline, CEA on follow-up of signet ring cancers remains essential, with 97% of secretors and 67% of non-secretors having rising CEA at recurrence.

On the other hand, a negative CEA in secretory patients is reassuring to both patients and doctors; less than 3% with normal CEA recur.

CEA elevation in signet cell colorectal cancer appears non-site specific, with most recurrences manifesting elevated CEA levels, irrespective of baseline secretor status.

## Data Availability

The data that support the findings of this study are available from the corresponding author upon reasonable request. However, restrictions apply to the availability of these data due to institutional regulations, and so they are not publicly available.
